# *Aucuba japonica* Extract and Aucubin Prevent Desiccating Stress-Induced Corneal Epithelial Cell Injury and Improve Tear Secretion in a Mouse Model of Dry Eye Disease

**DOI:** 10.3390/molecules23102599

**Published:** 2018-10-11

**Authors:** Wan Seok Kang, Eunsoo Jung, Junghyun Kim

**Affiliations:** 1 College Department of Oral Pathology, School of Dentistry, Chonbuk National University, Jeonju 54896, Korea; wskangjbnu@gmail.com; 2 Laboratory of Toxicology, Research Institute for Veterinary Science and College of Veterinary Medicine, Seoul National University, Seoul 08826, Korea; ozz79@snu.ac.kr

**Keywords:** *Aucuba japonica*, aucubin, cornea, dry eye, tear

## Abstract

Dry eye disease is affected by a broad range of causes such as age, lifestyle, environment, medication and autoimmune diseases. These causes induce tear instability that activates immune cells and promotes expression of inflammatory molecules. In this study, we investigated the therapeutic effects of an ethanolic extract of *Aucuba japonica* (AJE) and its bioactive compound, aucubin, on dry eye disease. The human corneal cells were exposed to desiccation stress induced by exposing cells to air, so that viability was decreased. On the other hand, pre-treatment of AJE and aucubin restored cell survival rate depending on the dose under the dry condition. This result was confirmed again by terminal deoxynucleotidyl transferase dUTP nick end labeling (TUNEL) staining. The mRNA expression of inflammatory molecules was reduced by the pretreatment of AJE and aucubin under the dry state. The therapeutic effects of AJE and aucubin were examined in the animal model for dry eye induced by unilateral excision of the exorbital lacrimal gland. Declined tear volumes and corneal irregularity in the dry eye group were fully recovered by the administration of AJE and aucubin. The apoptotic cells on the cornea were also decreased by AJE and aucubin. Therefore, this study suggests that administration of AJE can be a novel therapeutic for dry eye disease and that the pharmacological activities of AJE may be in part due to its bioactive compound, aucubin.

## 1. Introduction

Dry eye disease (DED) is a chronic pathogenic condition, which is characterized by a lack of tear production or an increase in tear evaporation, usually caused by ocular surface inflammation [1]. Most older people, but not exclusively them, are suffering DED; many of causes can affect the onset of it including intrinsic factors such as systemic medication, autoimmune disorders, or extrinsic factors such as low humidity of the environment, prolonged video display viewing and laser-assisted in situ keratomileusis (LASIK) surgery [2,3]. When the patients experience dysfunction of tear secretion, they already have a decreased function of secretory cells or glands resulting in a formation of the unstable tear film. Tear instability exerts the activation of stress-related signaling pathways of epithelial cells and resident inflammatory cells in the ocular surface, which induces the production of pro-inflammatory cytokines [4]. It may exacerbate the deficiency of tear production and worsen dry eye symptoms. Therefore, inhibition of inflammation and apoptosis can be a promising therapeutic strategy for DED.

*Aucuba japonica*, a perennial native plant of Korea and Japan, has been used as food and as a medicinal plant to treat several diseases including edema, abscess and gastrointestinal disorders [5]. The leaves have various chemical compounds such as aucubin and eucommiol-*O*-β-d-glucopyranoside [6]; in particular, aucubin is abundant, a small molecule that is known for anti-inflammatory and anti-oxidant effects [7,8], liver protection [9], gastroprotection [10], neuro-protection [8] and promoting angiogenesis [11]. Although *Aucuba japonica* and its bioactive ingredient, aucubin, have shown various pharmacological activities in vitro and in vivo, its pharmacological activities on DED still have not been evaluated. The purpose of this study was to examine the effects of an extract of *Aucuba japonica* and aucubin on desiccating stress-induced corneal epithelial cell injury in vitro and tear film instability in a mouse model induced by unilateral excision of the exorbital lacrimal gland and identify the possible mechanism of action.

## 2. Results

### 2.1. HPLC Analysis of AJE

**Figure 1:**
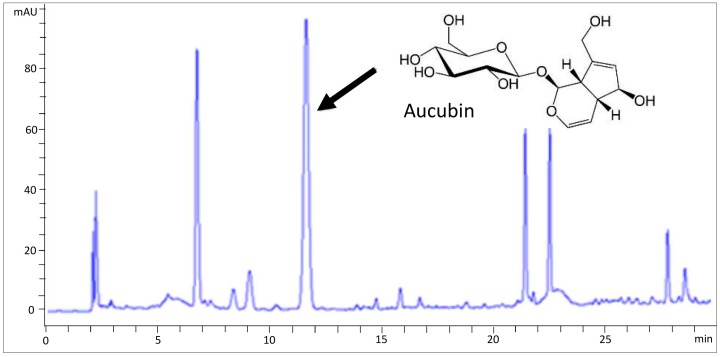
HPLC chromatographs of an extract of *Aucuba japonica*.

As shown in Figure 1, aucubin was found in AJE. The content of aucubin in AJE was 284.30 ± 1.54 mg/g (Table 1). According to the content of aucubin in AJE, we determined the doses of aucubin for in vitro and in vivo experiments.

### 2.2. AJE and Aucubin Improve Cell Viability against Desiccation Injury

**Figure 2:**
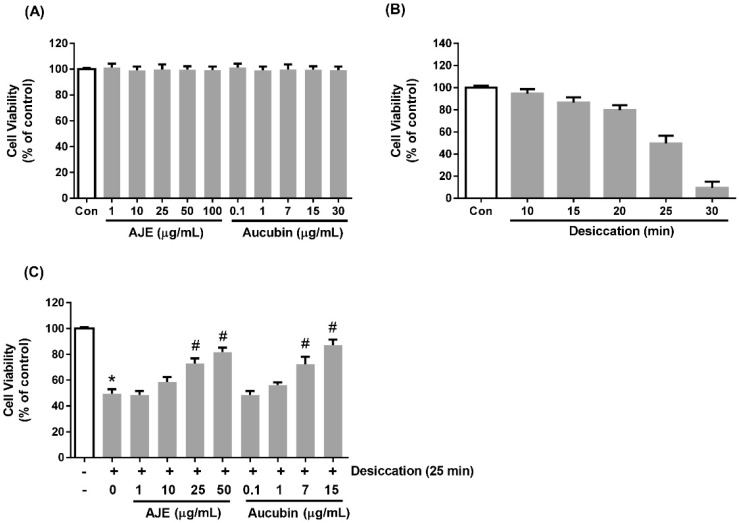
Effects of *Aucuba japonica* (AJE) and aucubin on the viability of human corneal epithelial cell. (**A**) The cell viability in the dose-dependent treatments of AJE and aucubin for 24 h. (**B**) The cell viability under the time-dependent desiccation. (**C**) The cells were pre-incubated with various concentrations of AJE and aucubin for 24 h and then exposed to air to induce desiccation stress. All of the viability is shown as % of control. The values in bar graph represent the mean ± SE. * *p* < 0.05 vs. control cell, ^#^ *p* < 0.05 vs. vehicle treated cell.

The cytotoxicity of AJE and aucubin to human corneal epithelial cells was examined by MTS assay. We tested 1 to 100 μg/mL range of AJE and 0.1 to 30 μg/mL range of aucubin. There was no cytotoxicity (Figure 2A). Desiccation was used as a stress corresponding to dry eye disease. The cells were exposed to air on the clean bench for 10 to 30 min, and 25 min was used as a moderate damage condition (Figure 2B). To investigate the preventive effects of AJE and aucubin against desiccation, the cells were pre-treated various concentrations of AJE and aucubin for 24 h. The cell viability was significantly recovered, more than 70% in both of 25 and 50 μg/mL of AJE- treated groups. Aucubin also restored the cell viability in a dose-dependent manner.

### 2.3. AJE and Aucubin Inhibit Apoptosis against Desiccation Stress

**Figure 3:**
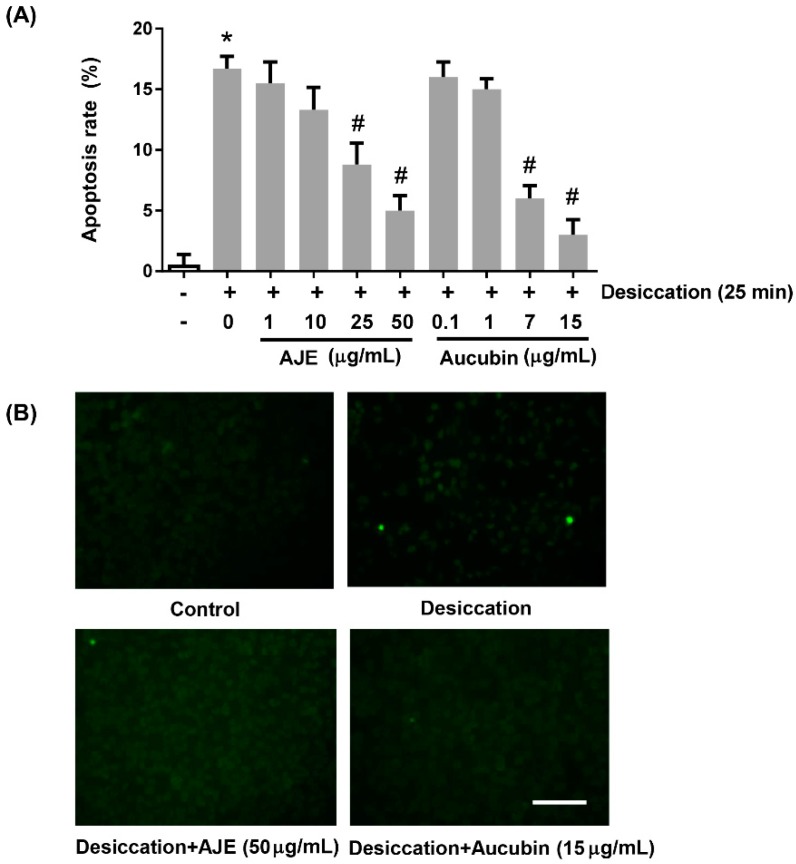
Effects of AJE and aucubin on apoptosis rate of human corneal epithelial cell under desiccation. The cell was pre-incubated with various concentrations of AJE and aucubin for 24 h and then exposed to air to induce desiccation stress. After that, the cells were fixed and performed TUNEL staining. (**A**) Apoptosis rate was calculated by comparing the ratio of TUNEL-positive cells to total cell number. (**B**) The images were taken under the fluorescence microscope after TUNEL staining. Scale bars = 100 μm. The values in bar graph represent the mean ± SE. * *p* < 0.05 vs. control cell, ^#^ *p* < 0.05 vs. vehicle treated cell.

To confirm the cytoprotective effects of AJE and aucubin more directly, we examined apoptosis assay. The apoptotic cells were detected by TUNEL staining. The dying cells were increased by desiccation process and the ratio of apoptotic cells was significantly decreased in both of 50 μg/mL AJE-treated group and 15 μg/mL aucubin-treated group (Figure 3A,B). These results gave us a possibility of use of AJE and aucubin as anti-dry eye disease reagent.

### 2.4. AJE and Aucubin Decrease mRNA of Inflammatory Cytokine Expression

**Figure 4:**
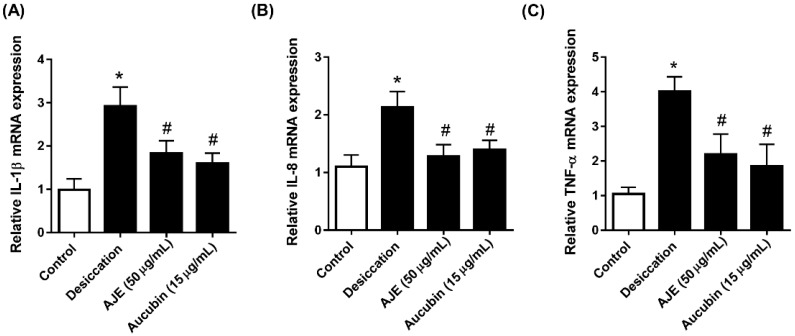
Effects of AJE and aucubin on mRNA level of inflammatory cytokines in human corneal epithelial cell. The cell was pre-incubated with 50 μg/mL of AJE and 15 μg/mL of aucubin for 24 h and then exposed to air to induce desiccation stress. Total RNA was extracted and the mRNA of (**A**) IL-1β, (**B**) IL-8 and (**C**) TNF-α were analyzed by real-time PCR. The data in bar graph are relative values compare to control and represent the mean ± SE. * *p* < 0.05 vs. control cell, ^#^ *p* < 0.05 vs. vehicle treated cell.

Dry eye disease is further encouraged by inflammation [4]. Therefore, we evaluated the expression of inflammatory genes to determine whether AJE and aucubin showed anti-inflammatory effects under dry conditions. The mRNA expression of IL-1β, IL-8 and TNF-α were significantly increased in the dry condition, and those were lowered by AJE and aucubin treatments (Figure 4A–C).

### 2.5. AJE and Aucubin Improve Tear Production and Corneal Irregularity in the DED Rats

**Figure 5:**
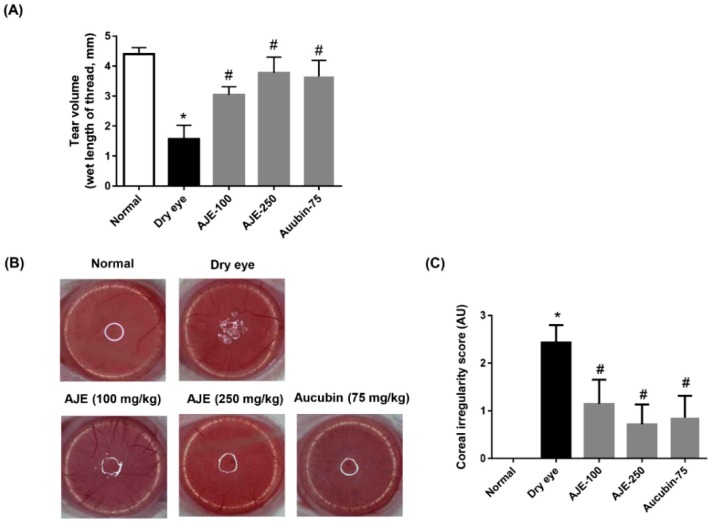
Effects of AJE and aucubin on tear secretion and corneal irregularity in dry eye rats. (**A**) Tear volume was measured using the phenol red thread test. Tear volume was expressed in millimeters of thread that became wet by the tear and turned red in color. (**B**) Reflected images of a white ring from the fiberoptic ring illuminator of a stereomicroscope. (**C**) Corneal irregularity was graded according to the number of distorted quarters in the reflected white ring as follows: 0, no distortion; 1, distortion in one quarter; 2, distortion in two quarters; 3, distortion in three quarters; 4, distortion in all four quarters; 5, severe distortion, in which no ring could be recognized. The values in bar graph represent the mean ± SE. n = 7. * *p* < 0.05 vs. normal rat, ^#^ *p* < 0.05 vs. vehicle treated dry eye rat.

In order to test AJE and aucubin on in vivo dry eye rat model, we induced experimental DED by surgical excision of the unilateral exorbital lacrimal gland. The AJE and aucubin were orally administered for 7 days. Tear production was determined using a phenol red-impregnated cotton thread and the wet lengths of thread indicated tear volume were compared. The experimental dry eye model showed significantly lower tear volume compared with the normal group, but those were recovered by AJE and aucubin treatments. In particular, the higher dose of AJE greatly recovered tear volume to almost normal. (Figure 5A). The corneal irregularity was assessed by taking pictures of the rat eye reflecting a white ring of light. The dry eye group showed an irregular circle shape of light, but AJE and aucubin treatments improved this change (Figure 5B). The scores of corneal irregularities showed that the dry eye group had a high score, but that AJE and aucubin treatments decreased it significantly (Figure 5C).

### 2.6. AJE and Aucubin Reduce Apoptotic Cells on the Cornea of Dry Eye Rats

**Figure 6:**
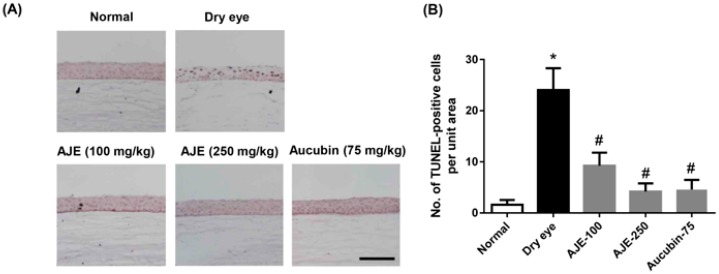
Effects of AJE and aucubin on the number of apoptotic cells in ocular surface of dry eye rats. (**A**) Apoptotic cells were stained by TUNEL assay. Scale bars = 100 μm. (**B**) TUNEL-positive cells were counted in the unit area. The values in bar graph represent the mean ± SE. n = 7. * *p* < 0.05 vs. normal rat, ^#^ *p* < 0.05 vs. vehicle treated dry eye rat.

As shown in the above data, in order to confirm whether the cytoprotective effect of AJE and aucubin actually work on an in vivo dry eye model, we performed TUNEL staining to detect apoptotic cells on cornea of rats. The apoptotic cells on the cornea were significantly increased in dry eye rats than those of normal rats, but those were reduced by AJE and aucubin treatments (Figure 6A,B).

## 3. Discussion

DED is a disorder of the tear film due to tear deficiency or excessive evaporation [12,13]. Numerous risk factors for DED have been reported, such as aging, gender, contact lens use, air-conditioning and visual display terminal work [14,15,16,17]. Deficiency of tears is caused by various pathophysiological mechanisms, including cell volume reduction, dysfunction to DNA repair systems, increased reactive oxygen species generation and increased apoptotic cell death in the ocular surface cells [18]. The most important core mechanism for DED is associated with hyperosmolarity of the tear film and ocular surface inflammation [19].

Current treatment strategies for dry eye disease are using artificial tears, lubricants and various ophthalmic drops, such as anti-inflammatory drugs, immunosuppressive agents and steroids. However, these treatments have focused on alleviating clinical symptoms rather than resolving the causes of DED. Therefore, in order to achieve a fundamental therapy of dry eye disease, we investigated the protective effect of a natural product, an extract of *Aucuba japonica*, that has traditionally been used for the treatment of burns, incised wounds and swelling. One of the major contents of the plant is aucubin, which has demonstrated multiple protective effects including anti-inflammation, anti-oxidation and tissue protection. We also examined whether aucubin is a major bioactive ingredient in the AJE.

Desiccation injury on cells used here was widely performed to discover therapeutic molecules or diseases progression mechanism [20,21,22]. AJE and aucubin treatments on corneal cell increased viability against desiccation injury in our data. It is the first preventive report using AJE and aucubin for the dry condition, even though a previous study showed similar data, that aucubin has a protective effect on H_2_O_2_ induced apoptosis [8]. This result was confirmed by TUNEL staining. As reported previously, the anti-inflammatory effect of aucubin was also detected by our AJE and aucubin treatments on dry injured cells. Aucubin prevents proinflammatory cytokine-induced inflammatory response by inhibition of NF-κB signaling [23,24]. Aucubin is also reported to regulate Bcl-2 family protein expression and inhibit apoptosis [8,25]. Ho et al. reported that aucubin protected against UVB-induced skin fibroblast activation by inhibition of the production of matrix metalloproteinase-1 [26]. Since inflammation and corneal epithelial cell injury are involved in the progression of DED, aucubin was predicted to have protective effects on DED. These anti-apoptotic and anti-inflammatory effects of AJE and aucubin may work synergistically to prevent desiccation injury, even though we did not confirm detailed mechanisms.

In the animal experiments, AJE and aucubin were orally administrated in the DED rats for 7 days. The tear volume and corneal irregularity were recovered almost to normal by AJE and aucubin treatment in our data. AJE and aucubin also decreased apoptotic cell injury of the cornea of dry eye rats. Our results suggest that the cytoprotective effects of AJE and its bioactive compound, aucubin, are mediated by its anti-apoptotic and anti-inflammatory activities. Inflammation acts as a major factor of DED. We did not test that AJE and aucubin directly induce tear secretion or expression of tear components, but we believe that it would be a good fundamental therapeutic, because AJE and aucubin clearly inhibit the loss of cells consisting of the cornea. Therefore, further study should be performed to confirm the effectiveness, as well as detailed therapeutic mechanisms and safety.

In conclusion, AJE and aucubin can inhibit corneal cell apoptosis induced by dry conditions. Progression of the dry eye disease is clearly suppressed by AJE and aucubin treatments, and dysfunction of tear secretion is also protected. Therefore, the current work demonstrates that AJE and aucubin may pave the pathway to treat dry eye disease.

## 4. Experimental Section

### 4.1. Preparation of Aucuba japonica Extract

The leaves and stems of cultivated *Aucuba japonica* were purchased from Namhae, Kyungsangnamdo, South Korea. The botanical identification was made by botanist Prof. Joo-Hwan Kim (Department of life science, Gachon University, Kyounggi-do, South Korea). The voucher specimen (No. JBNU-AJE) is deposited in the herbarium of the Korea Institute of Oriental Medicine (Daejoen, South Korea). To prepare the standardized *A. japonica* extract (AJE), 1 kg of the aerial parts of *A. japonica* was weighed accurately, and 30% ethanol (10 L) was added to the herb and extracted at 70 °C for 3 h. The extract solution was filtered and concentrated to get a 210 g extract. AJE was standardized by high-performance liquid chromatography (HPLC) using aucubin (Sigma, St. Louis, MO, USA) as a reference compound. According to the content of aucubin in AJE, we determined the doses of aucubin for in vitro and in vivo experiments.

### 4.2. Cell Culture and Cell Viability Assay

The human corneal epithelial cells (PCS-700-010, American Type Culture Collection, Manassas, VA, USA) were maintained in corneal epithelial cell basal medium containing growth supplements according to the manufacturer’s instructions (American Type Culture Collection, Manassas, VA, USA). Cell viability was examined using an MTS assay kit (Promega Corporation, Madison, WI, USA). Cells (1 × 10^4^ cells/well) were plated in 96-well plates. Cells at a density of 2 × 10^5^/well were treated with various concentrations of AJE (1–100 μg/mL) or aucubin (0.1–30 μg/mL). AJE and aucubin were dissolved in the culture medium. The content of aucubin in AJE was 284.30 ± 1.54 mg/g. According to the content of aucubin in AJE, we determined the doses of aucubin. Cell viability was measured 24 h after incubation. The results of the MTS assay were obtained by measuring absorbance using a microplate reader (Tecan Group Ltd., Männedorf, Switzerland) at 490 nm.

### 4.3. Cell Viability and Apoptosis under Desiccating Stress

Desiccating stress on the cell was induced after pre-incubation with AJE or aucubin for 24 h. To induce desiccating stress, the culture media was discarded and cells were exposed to different durations of desiccation (0, 10, 15, 20, 25, and 30 min). The viability of desiccated cell was measured by MTS assay as described in above. In order to detect apoptotic cells after desiccation, the cells were seed on the autoclaved coverslip on the plate. After pre-incubation with AJE for 24 h, the cells were exposed in a dry condition for 25 min and apoptotic cells were detected by TUNEL staining.

### 4.4. Real-Time PCR

Total RNA from the corneal epithelial cells was extracted using Trizol reagent (Invitrogen, Carlsbad, CA, USA), and cDNA was synthesized using M-MLV reverse transcriptase (Bioneer, Daejeon, Korea). PCRs were performed using an iQ5 Continuous Fluorescence Detector System (Bio-Rad, CA, USA), with 2xSYBR® Green PCR Master Mix (SYBR Premix Ex Taq TM, TaKaRa, Tokyo, Japan). All procedures were conducted according to the manufacturer’s instruction. The sequences of the primers were as follows: IL-1b sense 5′-AGCTACGAATCTCCGACCAC-3′; IL-1b antisense 5′-CGTTATCCCATGTGTCGAAGAA-3′; IL-8 sense 5′-ATGCTTTTGATCTGCACAGCTGCAC-3′; IL-8 antisense 5′-TGGTCCAGCAGGAATAACCCTCAG-3′; TNF-α sense 5′-CAGCCTCTTCTCCTTCCTGA-3′; TNF-α antisense 5′-GGAAGACCCCTCCCAGATAGA-3′; GAPDH sense 5′-ATGTTCGTCATGGGTGTGAA-3′, and GAPDH antisense 5′-GGTGCTAAGCAGTTGGTGGT-3′. The results were normalized to GAPDH.

### 4.5. Animal Experiments

Seven-week-old male SD rats (Orient Bio, Seoul, Korea) were deeply anesthetized with isoflurane (JW Pharmaceutical, Seoul, Korea). To induce experimental DED, the left exorbital lacrimal gland was surgically excised. At three days after surgery, the animals were divided randomly into four groups (n = 7/group): (1) vehicle-treated DED rat (DED); (2) 100 mg/kg AJE-treated DED rats (AJE-100); (3) 250 mg/kg AJE-treated DED rats (AJE-250); (4) 75 mg/kg aucubin-treated DED rats (Aucubin-75). AJE and aucubin were dissolved in distilled water and orally administered for 7 days. Seven rats in the normal group (NOR) received a sham operation. All procedures performed on the animals were approved by our Institutional Animal Care and Use Committee (IACUC approval No. 17-059).

### 4.6. Tear Volume Measurement

After a 7-day treatment of AJE, tear volume was measured using a phenol red thread (Zone Quick, FCI Ophthalmics, Pembroke, MA, USA). The cotton thread was placed in the lateral canthus for 30 s. The length of the color-changed thread was measured. Measurements of tear volume were obtained in both eyes.

### 4.7. Corneal Surface Irregularity

To evaluate desiccating stress-induced ocular surface change, the corneal surface irregularity was evaluated as previously described [18]. The irregularity of reflected light from the fiberoptic ring illuminator (SZ51; Olympus, Tokyo, Japan) on the corneal surface were scored as follows: 0, no distortion; 1, distortion in 1/4 quarter of the reflected ring shape; 2, distortion in 2/4 quarters; 3, distortion in 3/4 quarters; 4, distortion in 4/4 quarters; 5, severe distortion and no ring shape could be recognized.

### 4.8. TUNEL Staining

The apoptotic cells on the tissue were determined by terminal deoxyribonucleotidyl transferase (TdT)-mediated dUTP nick end labelling (TUNEL) staining according to the manufacturer’s instructions.

### 4.9. Statistical Analysis

Data in all of the tables and figures are presented as the mean ± standard error of the mean (SE). Significant differences were assessed using one-way analysis of variance (ANOVA) followed by Tukey’s multiple comparison test. Differences were considered statistically significant at *p* < 0.05.

## Figures and Tables

**Table 1:** Content of aucubin in the *Aucuba japonica* extract.

Compound	Content (mean ± SE, n = 3), mg/g (%)
Aucubin	284.30 ± 0.89 (28.4)
